# Carbonized Dual-Layer Balsa Wood Membrane for Efficient Oil–Water Separation in Kitchen Applications

**DOI:** 10.3390/membranes15060160

**Published:** 2025-05-24

**Authors:** Mamadou Souare, Changqing Dong, Xiaoying Hu, Junjiao Zhang, Juejie Xue, Quanjun Zheng

**Affiliations:** 1 National Engineering Laboratory for Biomass Power Generation Equipment, School of New Energy, North China Electric Power University, Beijing 102206, China; generalsouare1@gmail.com (M.S.); huxy@ncepu.edu.cn (X.H.); junjiexue@ncepu.edu.cn (J.X.); 2 State Key Laboratory of Alternate Electrical Power System with Renewable Energy Sources, North China Electric Power University, Beijing 102206, China; 3 School of Energy, Power and Mechanical Engineering, North China Electric Power University, Beijing 102206, China; zjunjiao@ncepu.edu.cn; 4 Beijing Aerospace Petrochemical EC and EP Technology Corporation Limited (BAEEC), Beijing 100176, China; zhengqj@calt11.cn

**Keywords:** carbonized balsa wood, oil–water separation, kitchen wastewater, hydrophobicity/hydrophilicity

## Abstract

Wood-based membranes have garnered increasing attention due to their structural advantages and durability in the efficient treatment of oily kitchen wastewater. However, conventional fabrication methods often rely on toxic chemicals or synthetic processes, generating secondary pollutants and suffering from fouling, which reduces performance and increases resource loss. In this study, an innovative bilayer membrane was developed from balsa wood by combining a hydrophilic longitudinal layer for water transport with a polydimethylsiloxane (PDMS)-impregnated carbonized transverse layer to enhance hydrophobicity, resulting in increased separation efficiency and a reduction in fouling by 98.38%. The results show a high permeation flux of 1176.86 Lm^–2^ h^–1^ and a separation efficiency of 98.60%, maintaining low fouling resistance (<3%) over 20 cycles. Mechanical tests revealed a tensile strength of 10.92 MPa and a fracture elongation of 10.42%, ensuring robust mechanical properties. Wettability measurements indicate a 144° contact angle and a 7° sliding angle with water on the carbonized side, and a 163.7° contact angle with oil underwater and a 5° sliding angle on the hydrophilic side, demonstrating excellent selective wettability. This study demonstrates the potential of carbonized wood-based membranes as a sustainable, effective alternative for large-scale wastewater treatment.

## 1. Introduction

Considering high efficiency, environmental protection, and sustainability, membrane technology has emerged as a key innovation for oil–water separation, offering numerous benefits such as high separation efficiency, reduced energy consumption, and operational simplicity [1,2,3,4,5]. This technology has the ability to selectively filter contaminants based on size, charge, or affinity. Considerable efforts have been dedicated to developing advanced membranes with unique structures and functions, utilizing various materials from polymers to advanced composites to enhance performance and treat oily wastewater [6,7,8]. For instance, Wu et al. developed liquid-infused aerogel membranes enabling on-demand emulsification and demulsification. These membranes, fabricated from aramid nanofibers and polyvinylpyrrolidone, achieve an oil–water separation factor of 99.97% and demonstrate stable performance over multiple cycles [6]. Similarly, Kadadou et al. developed polyethersulfone (PES) membranes functionalized with polydopamine and cerium metal–organic frameworks (Ce-MOFs), achieving high water flux (337 Lm^−2^ h^−1^) and an oil removal efficiency of 98% [9]. Additionally, Wang et al. demonstrated a CO_2_-responsive membrane that efficiently separates oil and water with over 99.9% efficiency, utilizing capillary force self-assembly to switch between hydrophobicity and hydrophilicity [10]. Despite these advances, many of the developed materials suffer from limited stability and require additional chemical treatments to maintain their performance. These treatments tend to result in complex, energy-intensive, and economically inefficient processes. Moreover, their low hydrophilicity, tendency to aggregate, and environmental and toxicological impacts drive research toward more sustainable, high-performance, and cost-effective alternatives.

In this regard, among recent developments, carbon-based membranes, particularly those made from activated carbon, carbon nanotubes (CNTs), or graphene oxide, have shown significant promise in handling complex oil–water mixtures. These materials offer excellent chemical stability, mechanical strength, and tunable surface properties, making them ideal for oil–water separation [11,12,13]. However, the single-layer membranes made from these materials often encounter challenges related to fouling, where the oil accumulates on the surface of the membrane, significantly reducing its efficiency over time [14,15,16]. This fouling issue not only leads to a decline in separation performance but also requires frequent maintenance or membrane replacement, further increasing operational costs. Scholars have highlighted these challenges, noting that while the chemical and mechanical properties of single-layer membranes are generally favorable, their long-term stability and resistance to fouling remain key concerns. Furthermore, the limited scalability of these materials, often due to high production costs or a reliance on synthetic processes, restricts their broader application in real-world scenarios.

To address these challenges, bilayer membranes have recently emerged as a promising alternative, integrating distinct layers with complementary functionalities—typically a hydrophilic layer to facilitate water permeation and a hydrophobic layer to repel oil. This architectural innovation enhances separation efficiency, reduces fouling susceptibility, and offers a platform for material synergy [17,18,19]. Notably, Li et al. developed a multifunctional polypolyvinylidene fluoride (PVDF)–fabric bilayer membrane featuring electrochemical self-cleaning capabilities. Their design achieved 90.36% dye rejection and >99% oil–water separation efficiency while maintaining a flux of 337.36 Lm^−2^ h^−1^ without external pressure. The incorporation of Fe-Co-loaded carbon nanotubes enabled regenerative performance at 6 V direct current [20]. While demonstrating excellent multifunctionality, this system requires continuous energy input and relies on environmentally persistent fluoropolymers. Similarly, Cao et al. engineered a unidirectional transport Janus membrane with a gradient pore structure, achieving ultrafast water penetration (0.05 s) and stable separation efficiency (92.5%) over multiple cycles. Their design, combining polyacrylonitrile/polyethylene glycol and polycaprolactone layers, maintained a flux exceeding 205 Lm^−2^ h^−1^ [21]. However, the batch electrospinning fabrication method and potential polyethylene glycol (PEG) leaching may limit scalability. Furthermore, Zhang et al. reported a breathable bilayer Janus membrane with exceptional moisture transport properties (9065 gm^−2^ d^−1^). Their thermoplastic polyurethane/polyacrylonitrile-dopamine system demonstrated directional water transport through engineered wettability gradients [22]. Although promising for textile applications, its oil–water separation capabilities remain unverified.

However, despite these advantages, there are still several challenges that need to be addressed. The fabrication of these membranes often relies on synthetic or costly materials, limiting their large-scale application [18,19,23]. Furthermore, issues such as layer delamination, interfacial instability, and poor mechanical strength can affect the long-term performance of double-layer membranes. These challenges underscore the need for new materials and fabrication techniques that can improve the stability, scalability, and cost-effectiveness of bilayer membrane technologies and biocompatibility.

In response to these challenges, this study proposes the development of an innovative bilayer membrane for oil–water separation via a simple and eco-friendly approach, utilizing wood as a base material. Wood, with its intrinsic microchannel structure, offers several unique advantages for membrane design. The natural vertical alignment of microchannels in wood facilitates efficient water transport through capillary action [24,25,26,27]. This characteristic could reduce flow resistance while improving overall separation efficiency, without requiring complex treatments to create porous structures as is often the case with synthetic membranes [24,27,28]. Furthermore, after carbonization, wood develops hydrophobic and oleophilic properties that are essential for effectively separating oils from wastewater [29,30]. Wood is also an abundant, renewable, and biodegradable resource, providing an environmentally friendly alternative to polymer- or carbon-based synthetic membranes [31,32,33]. This study aims to combine these advantages by developing a bilayer membrane, where an unmodified wood layer with vertical microchannels facilitates water transport and a carbonized wood layer enhances oil retention through its hydrophobic and oleophilic properties. By independently optimizing the properties of each layer, this design seeks to improve separation efficiency and reduce fouling. Moreover, the optimization of fabrication parameters, including the carbonization process and layer integration, will be evaluated to assess the membrane’s performance in terms of permeation flux, separation efficiency, and long-term stability. This novel membrane design offers the potential for an eco-friendly, cost-effective, and high-performance solution to the pressing challenge of oily wastewater treatment.

## 2. Experimental Section

### 2.1. Materials

Balsa wood, with densities ranging from 31 to 102.47 mg/cm^3^, was sourced from Ji’an Crab Kingdom Technology Co., Ltd., Ji’an, China, and chosen as the primary material. Polydimethylsiloxane (PDMS 184) was obtained from The Dow Chemical Company (Midland, TX, USA). A thermally conductive adhesive (Kafuter K-705) with a measured thermal conductivity of 2.0 ± 0.4 W/(m·K) was supplied by Guangdong Hengda New Materials Technology Co., Ltd. (Huizhou, China). Oils, including sunflower seed oil, used cooking oil, corn oil, and olive oil, were sourced locally. Additional reagents, such as methylene blue and Tween 80, were procured from local suppliers.

### 2.2. Membrane Fabrication

**Figure 1:**
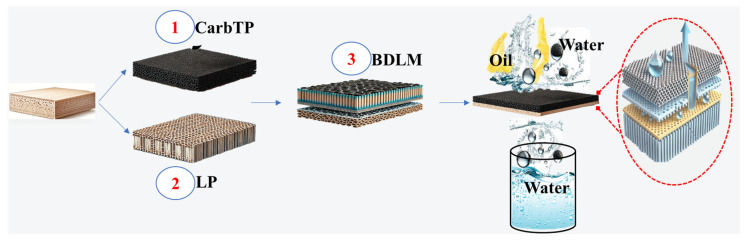
Schematic representation of the BDLM fabrication process for water–oil separation. Note: CarbTP: carbonized transverse piece; LP: longitudinal piece; and BDLM: balsa dual-layer membrane.

An inverted T-shaped dual-layer membrane (BDLM) optimized for oil–water separation was fabricated using natural balsa wood (Figure 1). Two distinct pieces were prepared, a longitudinal piece (LP) permeable to water and a denser transverse piece (TP) for oil retention, with target densities of 31 mg/cm^3^ and 102.47 mg/cm^3^, respectively. The TP was first preheated at 100 °C for one hour to prevent deformation during the carbonization process. Carbonization was performed in a tubular furnace under a nitrogen atmosphere, heating the TP to 800 °C at a ramp rate of 10 °C per minute and maintaining this temperature for two hours. This thermal treatment enhanced the hydrophobic properties of the TP while increasing its mechanical strength. After carbonization, the carbonized transverse piece (CarbTP) was treated with polydimethylsiloxane (PDMS) to impregnate its internal channels. This process was conducted under vacuum at 25 °C to ensure uniform penetration of the PDMS. Any excess PDMS on the surface was carefully removed, and the piece was cured at 60 °C for 1 h to stabilize its hydrophobic treatment.

The LP, measuring 0.1 cm × 2.3 cm × 2.4 cm, was prepared from raw balsa wood, retaining its natural hydrophilic properties and vertically aligned microchannels to facilitate water transport via capillary action. The CarbTP (0.1 cm × 2.2 cm × 2.3 cm) and the LP were assembled using a thermally conductive adhesive, forming a robust bond between the layers. The edges of the LP were trimmed to create a compact and streamlined inverted T-shaped structure, finalizing the BDLM and enhancing its efficiency for oil–water separation.

### 2.3. Characterization

The morphology of the membrane was analyzed using a scanning electron microscope (SEM, Hitachi SU4800, Hitachi High-Tech Corporation, Tokyo, Japan) to observe the surface features and internal structure of the two layers. High-resolution images allowed visualization of the vertically aligned microchannels in the hydrophilic layer and the horizontally aligned microchannels in the carbonized layer, as well as the interface between these two layers. This analysis assessed the integrity of the layers and their interaction, particularly the quality of the bond between the natural wood hydrophilic layer and the carbonized hydrophobic layer.

The functional groups present on the membrane surface were examined using Fourier-transform infrared spectroscopy (FTIR, Thermo Fisher Nicolet 6700, Thermo Fisher Scientific, Madison, WI, USA) in the range of 400–4000 cm^−1^. X-ray photoelectron spectroscopy (XPS, Thermo K-ALPHA, Thermo Fisher Scientific, Waltham, MA, USA) was used to study the chemical elements present on the membrane surface to confirm the introduction of specific chemical groups on the carbonized layer before and after carbonization.

### 2.4. Wettability Properties and Angle of Contact Measurement

The wettability of the dual-layer membrane was evaluated by determining water and oil contact angles using a goniometer (JC2000D, Shanghai-Zhongchen Power Technology Co., Ltd., Shanghai, China). A 5 µL droplet was placed on each layer, and the contact angles were measured. Results confirmed the hydrophilic nature of the unmodified wood layer (low water contact angles) and the hydrophobic/oleophilic properties of the carbonized PDMS-impregnated layer (high water contact angles). Measurements were also taken before and after several oil–water separation cycles. The variations in contact angles were analyzed to assess changes in surface properties, fouling resistance, and membrane durability.

### 2.5. Mechanical and Thermal Properties

The mechanical properties of the membrane were assessed using an electronic tensile testing device (WH-5000, Ningbo Weiheng Testing Instruments Co., Ltd., Ningbo, China). Tensile strength and break force were evaluated to test the structural stability of the membrane. The samples were subjected to a tensile force of 5 kN/100 N, and the maximum resistance was determined based on the membrane′s dimensions (length, width, and thickness) to ensure that the membrane could withstand mechanical stresses during separation cycles.

The thermal conductivity of the TP, the CarTP, and the BDLM was determined using an LFA 467 (LFA 467 HighTemp S, NETZSCH, Selb, Germany), a thermal diffusivity analyzer. The analysis was conducted using the flash thermal diffusivity method at a temperature of 200 °C, where a flash lamp was used to excite the samples. This method allowed for the evaluation of the membrane’s ability to manage thermal variations and reduce thermal losses, which is essential for the separation process.

### 2.6. Oil–Water Separation Performance

To assess the membrane’s separation performance, synthetic kitchen oil–water emulsions were created by mixing water with oil at a volumetric ratio of 1:1 and homogenized at 5000 rpm for duration of 5 min. The prepared emulsion was passed through the membrane under gravity. The volume of water collected in the permeate was measured to evaluate separation performance. The water flux (*j*, Lm^−2^ h^−1^) was calculated using the equation *j* = *V*/(*A* × *t*), where *V* is the volume of permeated water, *A* is the effective membrane area, and *t* is the filtration time. The separation efficiency (*η*, *%*) was calculated as *η* = (*ms*/*m*_0_) × 100, where *ms* is the volume of separated water and *m*_0_ is the initial water volume in the emulsion, as described in our previous work [5].

### 2.7. Fouling Resistance and Reusability Testing

The fouling resistance of the membrane was tested by performing several oil–water separation cycles. After each cycle, the membrane was cleaned with a 50:50 ethanol–water mixture to remove oil and restore performance. Flux and separation efficiency were measured after each cycle to track any performance decline. The flux recovery ratio (*F_R_*, %) and the fouling resistance (*R_F_*, %) were determined using the following Equations (1) and (2):

(1) $$F_{R}=\left(\frac{J_{n}}{J_{0}}\right)\times 100$$

(2) $$R_{F}=\left(\frac{J_{0}-J_{n}}{J_{0}}\right)\times 100$$

where *J*_0_ represents the initial flux and *J_n_* is the flux after the *n*-th cycle. A higher *F_R_* and lower *R_f_* indicate good antifouling behavior. The membrane’s reusability was tested over 20 consecutive cycles, with performance evaluated after each cycle. Cleaning with ethanol between cycles helped assess the long-term durability by monitoring changes in separation efficiency and flux.

## 3. Results and Analysis

### 3.1. Preparation of the Dual-Layer Membrane

The fabrication process of the BDLM highlights the functional synergy between the hydrophilic longitudinal piece (LP) and the hydrophobic transverse piece (TP), leveraging the anisotropic properties of balsa wood. Due to its natural anisotropy, wood exhibits higher efficiency in heat and mass transfer along its longitudinal direction compared to its transverse direction. To optimize water transport, the LP was sourced from low-density balsa wood (31 mg/cm^3^), characterized by thin cell walls and vertically aligned microchannels. These properties enable the LP to serve as an effective pathway for capillary-driven water transport. In contrast, the TP, cut from high-density balsa wood (~102.47 mg/cm^3^) with thicker cell walls, was selected for its superior structural stability and thermal conductivity, which are both essential for ensuring efficient separation and durability under operational conditions.

The TP underwent a carbonization process at high temperature, during which its color changed from pale yellow to black while retaining the honeycomb-like microstructure of natural wood. This structural retention ensures the preservation of its inherent anisotropic properties. Successful carbonization was confirmed by the emergence of characteristic graphitic peaks observed through Raman spectroscopy, indicating the formation of graphitic structures which are known to enhance both thermal conductivity and photothermal conversion efficiency. These properties are critical for improving the interfacial heat transfer within the membrane and facilitating efficient oil–water separation. The chemical changes induced by carbonization were further evidenced by the FTIR spectrum, which showed a significant reduction in the hydroxyl stretching peak (~3350 cm^−1^), indicating the loss of hydroxyl groups. This chemical transformation was corroborated by XPS analysis, which revealed a decreased oxygen-to-carbon ratio, signifying the elimination of oxygenated functional groups. While the carbonized TP (CarbTP) exhibited improved hydrophobicity with a water contact angle increasing to approximately 119°, this was insufficient to fully repel water infiltration under practical conditions.

**Figure 2:**
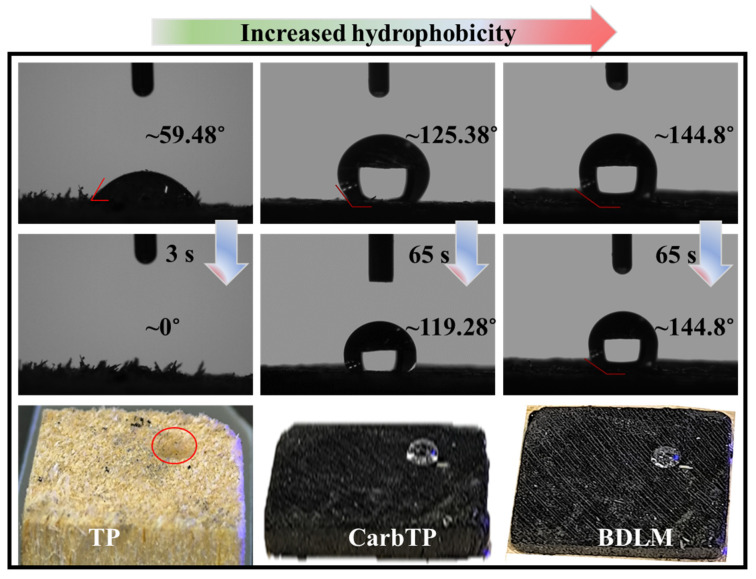
Contact angles of water on TP, CarbTP, and BDLM surfaces.

To address this limitation, the CarbTP was impregnated with hydrophobic polydimethylsiloxane (PDMS), filling its internal microchannels. This treatment significantly enhanced the hydrophobic properties of the transverse layer, achieving a water contact angle of approximately 144.8° (Figure 2). This increase in hydrophobicity ensures the effective prevention of water penetration into the hydrophobic layer during the separation process. Additionally, the PDMS treatment further improved the solid-phase thermal conduction of the CarbTP without altering its inherent heat transfer pathways, thereby ensuring efficient interfacial heat transfer and robust performance.

By integrating the hydrophilic LP with the hydrophobic and oleophilic PDMS-treated CarbTP, the dual-layer membrane was engineered to optimize the separation of oil–water emulsions, leveraging the complementary properties of both layers. This design ensures effective water transport while preventing oil penetration, making it well-suited for the treatment of kitchen oil wastewater.

### 3.2. Morphological and Structural Characterization

**Figure 3:**
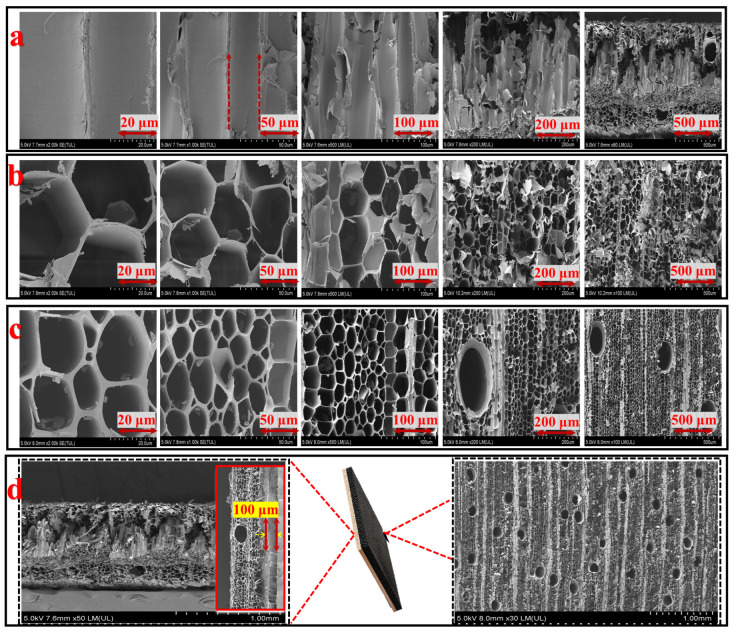
SEM images of cross-sections of the LP (**a**) and TP before (**b**) and after carbonization (**c**). A top-view SEM image showing the compact bonding interface between the LP and CarbTP layers (**d**).

The morphological characteristics of the dual-layer membrane, illustrated in Figure 3, exhibit marked structural differences between the LP and TP layers before and after carbonization, as revealed by scanning electron microscopy (SEM).

The LP (Figure 3a) exhibits vertically aligned microchannels with relatively smooth walls, which facilitate water transport through capillary action. This organized pore architecture enhances water flux by minimizing flow resistance and promoting efficient liquid movement.

In contrast, the TP before carbonization (Figure 3b) shows a dense and heterogeneous structure with irregularly distributed larger pores. This structural heterogeneity significantly limits the TP layer′s effectiveness as a hydrophobic barrier. Larger and interconnected pores promote internal connectivity, increasing the risk of oil infiltration through the membrane. Moreover, the lack of structural homogeneity compromises the membrane′s ability to selectively separate oil from water.

Following carbonization at 800 °C under a nitrogen atmosphere, the TP (CarbTP) undergoes a distinct microstructural transformation, characterized by thickened walls and well-defined pores (50 μm) (Figure 3c). This transformation results from the thermal degradation of organic components and leads to enhanced mechanical stability and intrinsic hydrophobicity due to the partial graphitization of the material. The increase in surface roughness and the reduction in pore connectivity further contribute to the selective oil–water separation performance of the membrane.

The top-view SEM image (Figure 3d) reveals a compact bonding interface approximately 100 μm thick between the LP and CarbTP layers, resulting in a total membrane thickness of 3 mm. This thin yet robust interface is expected to enhance the transfer of materials between the layers, ensuring that the mechanical stability during filtration operations. This morphological contrast confirms the effectiveness of the carbonization treatment in optimizing the oil–water separation performance of the dual-layer system by enhancing TP layer hydrophobicity and mechanical strength while preserving LP layer water transport capacity.

### 3.3. Chemical Analysis of the Membranes

**Figure 4:**
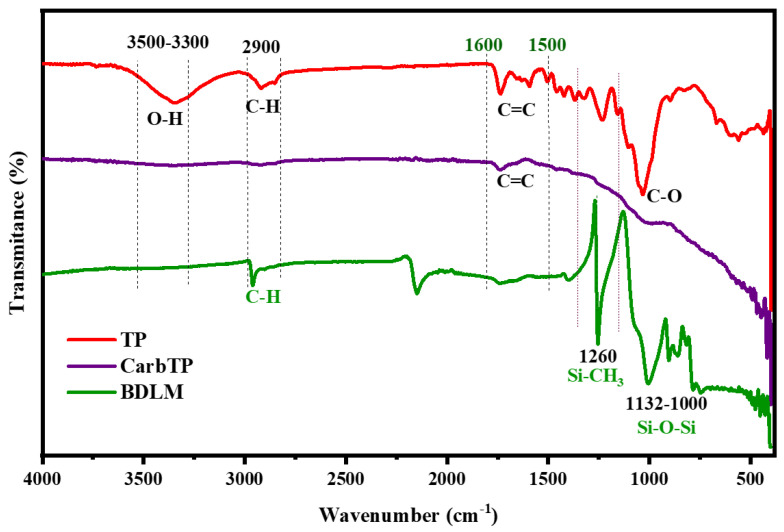
FTIR spectra of TP; CarbTP; and BDLM.

Figure 4 illustrates the FTIR spectra of wood samples before carbonization (TP), after carbonization (CarbTP), and after carbonization followed by a PDMS treatment of the BDLM. The spectrum of the TP sample reveals characteristic bands of the organic components of wood, primarily cellulose, hemicellulose, and lignin. A broad band around 3300–3500 cm^−1^ is associated with the stretching vibrations of O-H bonds, indicating the hydrophilic nature of the wood polymers [34]. The bands around 2900 cm^−1^ correspond to the C-H vibrations of methyl and methylene groups, while the peaks at 1600 cm^−1^ and 1500 cm^−1^ are attributed to aromatic C=C vibrations in lignin [34,35]. The band at 1030–1100 cm^−1^ corresponds to C-O stretching vibrations in carbohydrates, confirming the presence of polysaccharide structures.

After carbonization of the CarbTP, the spectrum shows a significant reduction in the bands associated with organic functional groups. The disappearance of O-H and C-H bands indicates the thermal degradation of cellulose, hemicellulose, and lignin, while the appearance of a broad band around 1600 cm^−1^ is attributed to the C=C vibrations in amorphous graphitic structures. This transformation reflects the conversion of organic components into amorphous carbon. With the addition of PDMS to the membrane (BDLM), new bands appear, notably at 2960 cm^−1^ and 1260 cm^−1^, corresponding to the C-H and Si-CH_3_ vibrations of PDMS, respectively, while a band at 800 cm^−1^ confirms the Si-O-Si vibrations of silicone. These results demonstrate that PDMS was successfully deposited on the surface to enhance the hydrophobic properties of the membrane without significantly altering the internal structure of the carbonized material. This surface modification makes the material suitable for oil–water separation applications.

### 3.4. XPS Characterization

**Figure 5:**
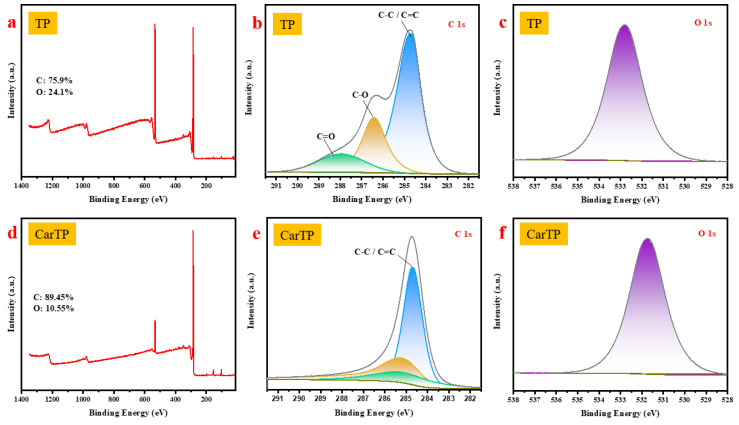
(**a**) Overview XPS spectrum of TP; (**b**,**c**) detailed XPS spectra corresponding to the C1s and O1s regions of TP; (**d**) XPS survey spectrum and (**e**,**f**) high-resolution XPS of C1s and O1s spectra of the CarbTP.

The XPS analysis highlights significant chemical differences between the natural balsa wood TP and its carbonized form CarbTP. The TP sample (Figure 5a–c) shows a composition primarily dominated by carbon (C) and oxygen (O), reflecting the presence of functional groups such as C-O and C=O, which are characteristic of cellulose, hemicellulose, and lignin. The spectra show C1s peaks (~284.8 eV) for C-C/C=C bonds, C-O (~286.5 eV) for hydroxyl and ether groups, and C=O (~288 eV) for carbonyls or esters, respectively. The O1s spectrum highlights peaks at ~531.5 eV for C-O and ~530.1 eV for C=O, confirming an abundance of oxygenated bonds [36]. These results indicate a hydrophilic structure rich in functional groups, typical of natural wood.

After carbonization (Figure 5d–f), the CarbTP sample shows significant compositional changes, with an increase in carbon content (89.45%) and a marked reduction in oxygen (10.55%). The C1s spectrum (Figure 5e) is dominated by a strong peak at 284.85 eV, indicating the prevalence of C-C/C=C bonds, characteristic of a more graphitic structure. Simultaneously, the O1s spectrum (Figure 5f) shows a substantial decrease in the intensity of oxygenated groups, with a residual peak at 532.82 eV corresponding to traces of C-O or O=C. These changes reflect the degradation of hydrophilic functional groups and the formation of a more graphitic, hydrophobic, and thermally stable material, making it suitable for advanced applications such as energy storage and filtration.

**Figure 6:**
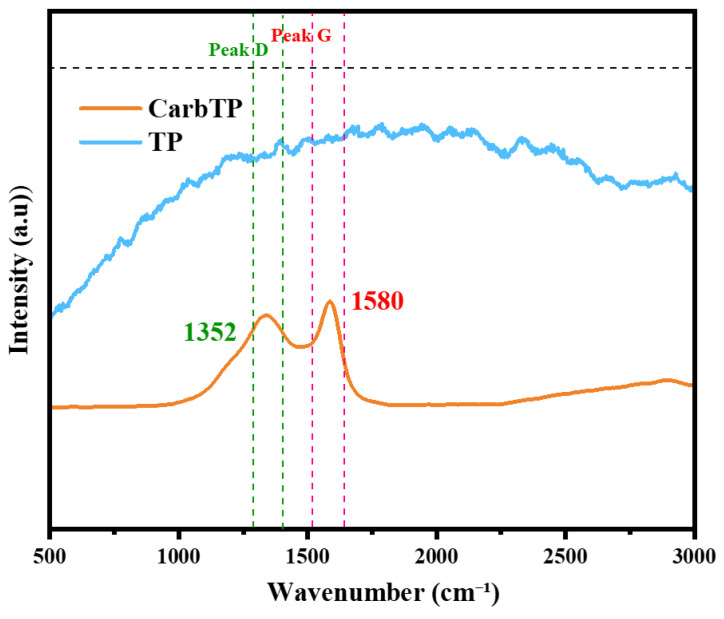
Raman spectra of TP and CarbTP.

Figure 6 illustrates the Raman spectra of wood TP samples before and after carbonization (CarbTP—carbonized at 800 °C). Before carbonization, the spectra exhibit characteristic bands of the main organic components of wood, primarily lignin and cellulose. The peaks located around 1375 cm^−1^ and 1600 cm^−1^ correspond to CH/OH deformation vibrations and aromatic C=C vibrations, respectively, which are characteristic of a structure rich in complex organic compounds. These bands reflect the hydrophilic nature and porous structure of raw wood.

After carbonization, the spectra reveal significant structural transformations. The characteristic organic bands disappear, replaced by two dominant peaks, the D band (~1350 cm^−1^), indicative of defects in the amorphous carbon structure, and the G band (~1580 cm^−1^), associated with C=C vibrations in sp^2^ structures [37]. These spectral changes confirm the thermal degradation of cellulose and lignin, leading to the formation of amorphous carbon, which is typical of pyrolyzed materials. The carbonization process under a N_2_ atmosphere at 800 °C has thus transformed the wood into a mechanically reinforced hydrophobic material, which is suitable for oil–water separation applications. The *ID*/*IG* ratio in the carbonized samples further reflects the degree of disorder in the resulting carbon network. This spectral evolution validates the structural modifications anticipated through the thermal treatment.

### 3.5. Optical Properties of TP and CarbTP: Transmittance, Reflectance, and Absorbance Analysis

The photothermal conversion performance of the samples was evaluated by analyzing the optical properties of the transverse natural piece (TP) and carbonized transverse piece (CarbTP), followed by the introduction of PDMS, using an UV–Vis spectrophotometer. These optical properties, including transmittance, reflectance, and absorbance, are crucial for understanding the photothermal conversion efficiency and the role of the material in oil–water separation [38,39]. Transmittance is essential for evaluating the material′s ability to allow light and water to pass through, while reflectance and absorbance directly influence the material′s thermal management and phase-separation efficiency. The differences observed between the TP and CarbTP highlight the impact of carbonization and PDMS treatment on membrane performance, particularly for applications that require efficient thermal regulation [40].

**Figure 7:**
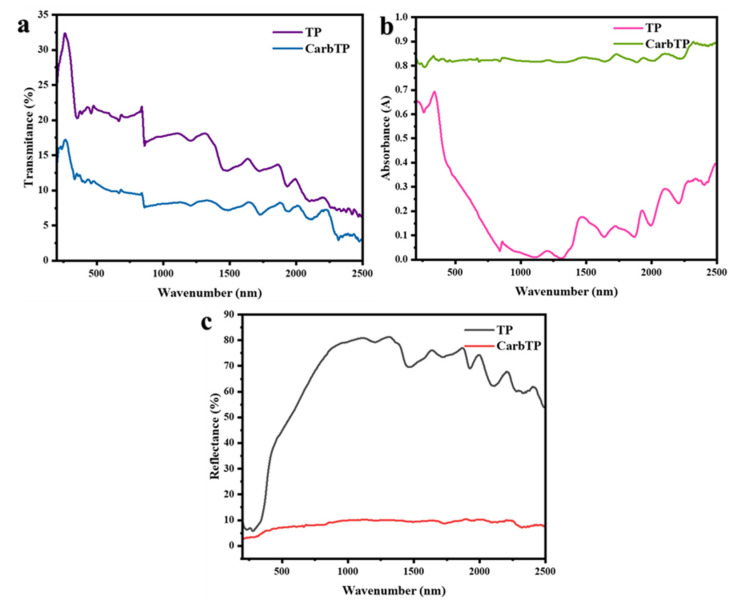
Transmittance (**a**); reflectance (**b**); and absorbance (**c**) spectra of TP and CarbTP.

As shown in Figure 7a, the TP exhibits a relatively high transmittance of 21.06% in the visible spectrum (400–700 nm), attributed to its porous and hydrophilic structure, which promotes light transmission and reflection, facilitating efficient water transport and supporting oil–water separation. In contrast, the CarbTP demonstrates a significantly lower transmittance of 10.52% due to the formation of graphite domains during carbonization, enhancing light absorption and enabling effective light-to-heat conversion with an absorbance of 82.23% (Figure 7b). This high absorbance allows the CarbTP to generate localized heat at the oil–water interface, reducing oil viscosity and destabilizing emulsions, thereby enhancing phase separation. The introduction of PDMS onto the CarbTP, forming the BDLM, maintains a high absorbance of 80.25%, indicating that PDMS does not significantly hinder light absorption while adding hydrophobic properties. Reflectance measurements (Figure 7c) further highlight the distinct roles of the TP and CarbTP; the TP exhibits an average reflectance of 49.08%, minimizing heat generation and enabling efficient water transport, whilst the CarbTP shows a much lower reflectance of 7.25% due to its rough surface and graphite domains, which, combined with its high absorbance, promote localized heat generation and emulsion destabilization. The high absorbance of both the CarbTP and BDLM is crucial for efficient photothermal conversion as the absorbed light energy reduces oil viscosity and accelerates phase separation, enhancing the membrane’s effectiveness in oil–water separation applications.

### 3.6. Thermal Conductivity of the BDLM

To evaluate the influence of structural modifications on thermal behavior, the thermal properties of the non-carbonized transverse piece (TP), the carbonized transverse piece (CarbTP), and the BDLM were systematically analyzed. This investigation focuses on understanding how carbonization and PDMS treatment affect thermal conductivity, thermal diffusivity, density, and specific heat capacity—key parameters for heat-assisted oil–water separation applications.

The thermal conductivity (*k*) shows a significant increase from 0.018 W/(m·K) in the TP to 0.081 W/(m·K) in the CarbTP, representing an enhancement by a factor of approximately 4.5. This enhancement is ascribed to the formation of a dense carbon-rich matrix during carbonization, which facilitates more efficient heat transfer by reducing thermal resistance. The BDLM exhibits a similar thermal conductivity of 0.080 W/(m·K), maintaining the thermal benefits of CarbTP while integrating the hydrophilic water-transport properties of the longitudinal piece (LP). The impregnation of PDMS into the CarbTP contributes to the structural stability of the membrane while having minimal impact on thermal conductivity due to the intrinsically low *k* values of PDMS. As a result, the modified TP evolves into a thermally efficient material, and the BDLM optimally balances heat dissipation and water transport functions.

In terms of the thermal diffusivity (*α*), a decreasing trend is observed—from 0.248 mm^2^/s in the TP to 0.224 mm^2^/s in the CarbTP, and further to 0.120 mm^2^/s in the BDLM. This reduction correlates with an increase in material density of 0.102 g/cm^3^ (TP), 0.136 g/cm^3^ (CarbTP), and 0.918 g/cm^3^ (BDLM). Although lower thermal diffusivity indicates slower heat propagation, the increased density compensates by enhancing thermal conductivity and sustaining effective heat dissipation.

**Figure 8:**
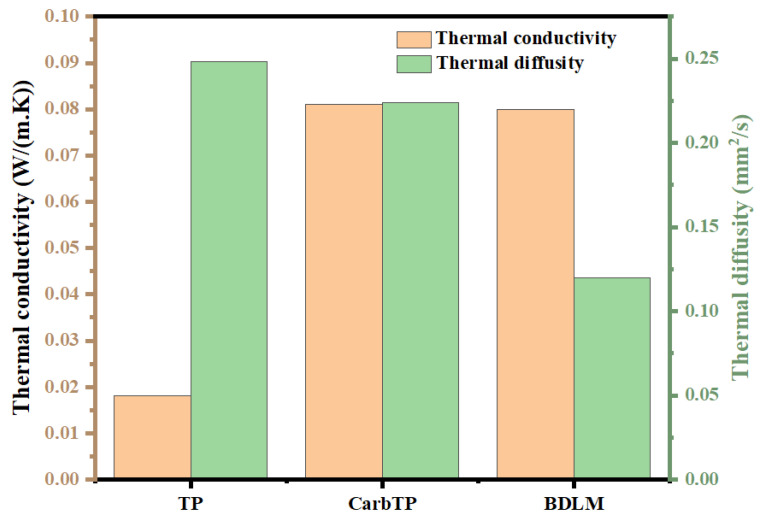
Thermal conductivity and diffusivity of TP, CarbTP, and BDLM.

The specific heat capacity (*C_p_*) also varies across the samples, rising from 0.694 J/(g·K) in the TP to 2.654 J/(g·K) in the CarbTP, and stabilizing at 0.729 J/(g·K) in the BDLM. These changes suggest a capacity for improved thermal energy storage, particularly in the carbonized form. Figure 8 summarizes these trends, illustrating the interdependence of the thermal diffusivity, density, and specific heat capacity across the different materials.

Overall, carbonization significantly enhances the thermal properties of the transverse piece, enabling its application in heat-assisted processes. The BDLM further integrates the high thermal conductivity of the CarbTP with the directional water transport capability of the LP, whilst also benefiting from PDMS-induced structural reinforcement. This synergy enables the BDLM to serve as an efficient and multifunctional platform for oil–water separation under thermal gradients.

### 3.7. Mechanical Properties Analysis

**Figure 9:**
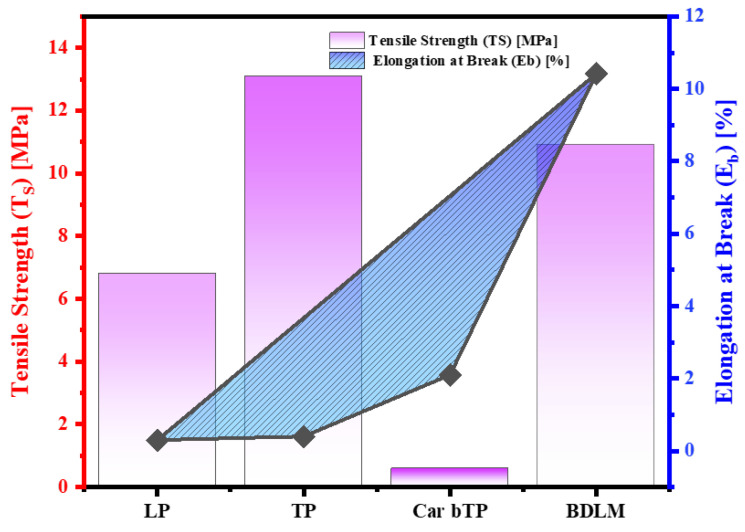
Mechanical properties of LP, TP, CarbTP, and BDLM.

The mechanical properties of the LP, TP, CarbTP, and BDLM samples were studied through tensile strength tests to evaluate their anisotropic mechanical behavior and suitability for specific applications such as oil–water separation. As illustrated in Figure 9, the results show that the TP exhibits the highest tensile strength (13.09 MPa) among the samples, attributed to its naturally dense and transverse structure. In contrast, the LP, while less strong (6.80 MPa), benefits from its longitudinal structure optimized for hydrophilic transport. The CarbTP, derived from the carbonization of the TP, experiences a significant decrease in tensile strength (0.60 MPa) but gains ductility, with an increased elongation at break (2.1%). This reflects a transition to a more flexible structure, favoring thermal and hydrophobic applications.

The BDLM combines the properties of the LP and CarbTP, offering significant tensile strength (10.92 MPa) and high flexibility (10.42% elongation at break). This highlights the complementarity of the layers in the dual-layer design, ensuring mechanical and functional stability in complex environments. Figure 9 presents a comparison of the tensile strength and elongation at break of all specimens, demonstrating the mechanical anisotropy of the individual components (LP, TP, and CarbTP) and the synergistic properties of the BDLM. The BDLM shows intermediate tensile strength and high flexibility, combining the advantages of both layers.

These results confirm that the BDLM, through its layered design and material treatments, achieves an optimal balance between rigidity and flexibility, making it suitable for demanding applications such as oil–water separation.

### 3.8. Mechanism of Transport and Separation

The transport and separation mechanism of oil and water through the BDLM relies on the complementary hydrophilic and hydrophobic properties of its two distinct layers. The bottom layer, composed of untreated hydrophilic natural wood, features vertically aligned pores (~50 μm) that facilitate rapid water transport via capillarity, governed by Darcy’s law [41], which is as follows:

(3) $$\;Q=-k\cdot A\cdot \left(\frac{\Delta P}{\mu \cdot L}\right)$$

where *k* represents permeability, *A* the surface area, Δ*P* the pressure difference, *L* is the thickness of the membrane, and *μ* is the dynamic viscosity of the fluid. This hydrophilic layer not only maximizes water flow but also acts as a natural barrier to oil infiltration, leveraging the capillary pressure created by the pore structure.

The top layer (CarbTP), carbonized at 800 °C under a nitrogen atmosphere, incorporates graphitic structures that enhance its hydrophobic properties, achieving a WCA of ~144.8° after PDMS impregnation. Its horizontally aligned microchannels (~30 μm) promote rapid water flow by exploiting the capillary effect, allowing water droplets to pass through this layer effortlessly under the influence of surface tension and adhesion forces, ultimately reaching the hydrophilic lower layer. At the same time, this structure effectively prevents oil infiltration by retaining it on the surface. Due to the difference in wettability between water and oil, along with the high surface tension of oil, its passage into the hydrophilic lower layer is blocked, ensuring efficient separation. This hydrophobic behavior is described by Young’s equation [42], which is as follows:

(4) $$cos\theta =\left(\frac{\gamma_{SV}-\gamma_{SL}}{\gamma_{LV}}\right)$$

where *γ_SV_*, γ*_SL_*, and *γ_LV_* indicate the surface energies of the solid–vacuum, solid–liquid, and liquid–vacuum interfaces, respectively, and *θ* is the contact angle formed between the liquid droplet and the solid surface, which characterizes the wettability of the surface.

**Figure 10:**
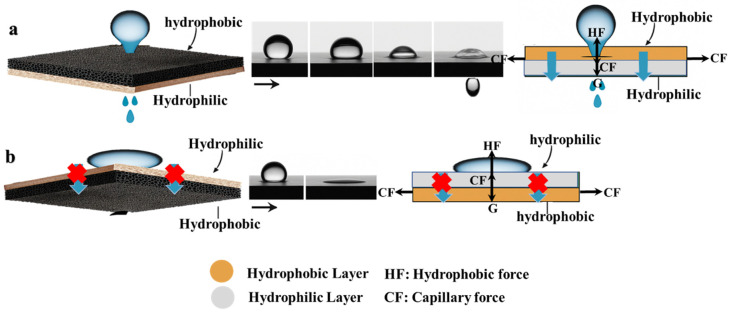
The mechanism of directional water transport: (**a**) from the hydrophobic side and (**b**) from the hydrophilic side.

The interplay between gravitational force (G), capillary force (CF), and hydrophobic force (HF) governs the selective liquid transport process. When a water droplet contacts the hydrophobic surface (Figure 10a), HF initially resists penetration. However, as G increases, the droplet overcomes this barrier and moves through vertical microchannels to reach the hydrophilic layer, where the combined action of G and CF facilitates absorption and transport [43,44,45]. Conversely, oil droplets, due to their lower surface tension and higher viscosity, remain on the hydrophobic layer, preventing contamination of the underlying structure (Figure 10b).

At the bilayer interface (~100 μm), SEM analysis confirms a robust bonding structure that ensures mechanical stability and efficient thermal management. The anisotropic thermal properties of the membrane reduce energy dissipation, enhancing separation efficiency while maintaining low hydraulic resistance to prevent clogging. This design enables long-term operation in complex wastewater environments, such as emulsified kitchen wastewater treatment, by effectively exploiting the distinct properties of each layer.

### 3.9. Special Wetting Properties Durability

**Figure 11:**
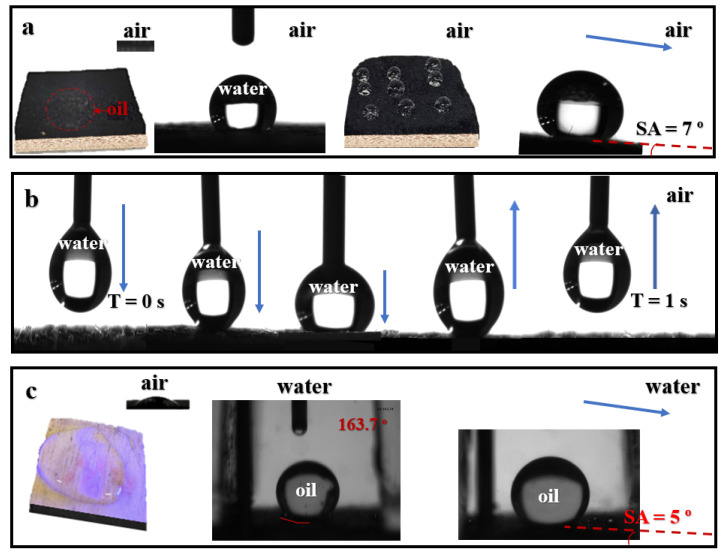
Wettability of the wood membrane: (**a**) images showing the static water contact angles and the behavior of water and oil droplets on the front side of the balsa wood membrane; (**b**) dynamic water contact angle images and the interaction of water and oil droplets with the front side of the membrane; and (**c**) static underwater oil contact angle images and the behavior of oil and water droplets on the back side of the membrane.

Wettability tests were conducted to evaluate the interaction of water and oil droplets with the composite membrane, consisting of the carbonized piece (TP) and the untreated piece (LP). The results, presented in Figure 11, demonstrate a remarkable asymmetrical wetting behavior on the both sides of the membrane.

In air, as shown in Figure 11a, an oil droplet placed on the upper surface (carbonized side) of the membrane is rapidly absorbed due to its superoleophilic properties. Conversely, a water droplet adopts a spherical configuration on the identical layer, displaying a notable water contact angle (WCA) of 144.8° and a minimal water sliding tendency (WSA) of 7°, confirming its good hydrophobicity. Furthermore, as illustrated in Figure 11b, the water droplet maintains its spherical form even when preload and release pressures are applied, demonstrating excellent water adhesion resistance.

On the lower surface of the membrane (untreated wood side), opposite behaviors are observed. As depicted in Figure 11c, water rapidly infiltrates through the vertically aligned microchannels of the natural wood, promoting capillary action. Simultaneously, an oil droplet placed on this side underwater remains spherical (supported by Video S1), with an oil contact angle (OCA) of 163.7° and an oil sliding angle (OSA) of 5°. This confirms the underwater superoleophobic nature of the untreated wood side.

This asymmetrical behavior offers significant practical advantages [46]. During the separation of oily wastewater, as schematically illustrated in Figure 11a,b, the hydrophilic and underwater superoleophobic lower surface prevents oil contamination and clogging of the wood’s microchannels. Concurrently, the hydrophobic and superoleophilic upper surface ensures efficient oil management. This bifunctional design maximizes separation efficiency while maintaining membrane durability.

#### 3.9.1. Separation Oil–Water Emulsified Performance

**Figure 12:**
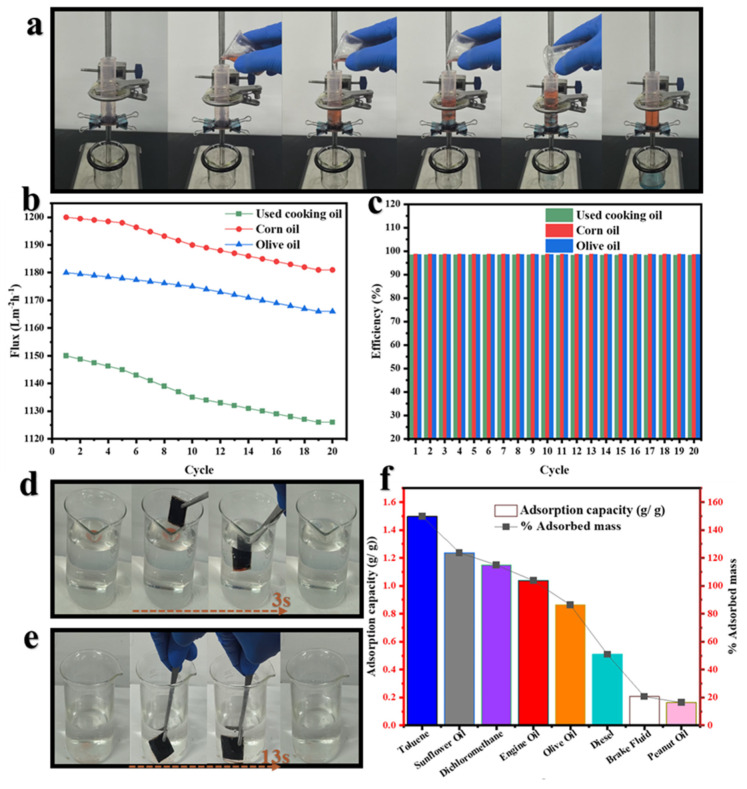
Oil–water separation experiment: (**a**) filtration process showing oil retention and water recovery; (**b**) oil–water filtration flux; (**c**) separation efficiency; (**d**) adsorption of floating oils on the water surface; (**e**) rapid adsorption of submerged oils at the water′s bottom; and (**f**) adsorption capacities and mass percentage of adsorbed oils.

The oil–water separation process using the BDLM is illustrated in Figure 12. The membrane achieved excellent oil–water separation performance, attributed to the synergistic behavior of its structural components. During filtration tests, oils were efficiently separated due to the synergistic action of the hydrophilic longitudinal piece (LP), which facilitated rapid water transport through capillary action, and the superhydrophobic transverse piece (CarbTP), which effectively blocked and coalesced oil droplets. Figure 12a illustrates the filtration process, supported by Video S2, which clearly shows water flowing through the membrane while oil is retained on the CarbTP surface.

The separation efficiency was evaluated using various types of oil, and the results revealed consistently high efficiency. The average filtration flux recorded for used cooking oil, corn oil, and olive oil were approximately 1166.86 Lm^−2^ h^−1^, 1189.98 Lm^−2^ h^−1^, and 1173.73 Lm^−2^ h^−1^, respectively, with an average separation efficiency of 98.38%, 98.77%, and 98.66% (Figure 12b,c). For the overall dataset, the mean filtration flux was 1176.86 Lm^−2^ h^−1^ and the mean separation efficiency was 98.60%. A comparison with existing literature (Table 1) shows that the BDLM exhibits superior flux rates compared to most previously reported systems while maintaining similarly high separation efficiencies.

After 20 consecutive filtration cycles, a slight flux reduction of about 2.09% was observed, attributed to minor oil accumulation in the microchannels. The separation efficiency remained high (>98%) for all tested oils, with the maximum performance recorded for corn oil (98.77%). These results suggest that the membrane enables efficient oil–water separation through filtration, exhibiting minimal flux reduction over multiple cycles.

To evaluate the adsorption ability of the prepared membrane in the water–oil separation process, the following eight types of oils and organic solvents with varying polarities were assessed: toluene, dichloromethane, brake fluid, peanut oil, engine oil, diesel, olive oil, and sunflower oil. The results demonstrated rapid absorption of both floating (Figure 12d) and submerged oils (Figure 12e), with capture times of approximately 3 s for floating oils and less than 13.2 s for submerged oils (supported by Videos S3 and S4, respectively). The maximum adsorption capacity was observed for toluene (1.50 g/g, 149.89% adsorbed mass), followed by sunflower oil (1.24 g/g, 123.75%) and dichloromethane (1.15 g/g, 114.97%). In contrast, more viscous oils such as peanut oil (0.16 g/g, 16.35%) and brake fluid (0.21 g/g, 20.61%) exhibited lower adsorption capacities. These results are illustrated in Figure 12e, which presents the adsorption capacities of the various solvents and oils tested.

Reusability tests over 20 consecutive cycles confirmed the durability and stability of the membrane, with minimal performance loss for both filtration and adsorption. These results highlight the exceptional efficiency of the carbonized T-shaped membrane for treating oily wastewater, combining high separation efficiency, low fouling, and adaptability to oils of varying viscosities.

#### 3.9.2. Fouling Resistance

The performance of the BDLM was evaluated over 20 consecutive oil–water separation cycles using the following three types of oils: used cooking oil, corn oil, and olive oil. The initial water fluxes were 1150 Lm^−2^ h^−1^ for used cooking oil, 1200 Lm^−2^ h^−1^ for corn oil, and 1180 Lm^−2^ h^−1^ for olive oil. After 20 cycles, the final flux values slightly decreased to 1126 Lm^−2^ h^−1^, 1181 Lm^−2^ h^−1^, and 1166 Lm^−2^ h^−1^, corresponding to flux recoveries (*F_R_*) of 97.91%, 98.42%, and 98.81%, respectively.

**Figure 13:**
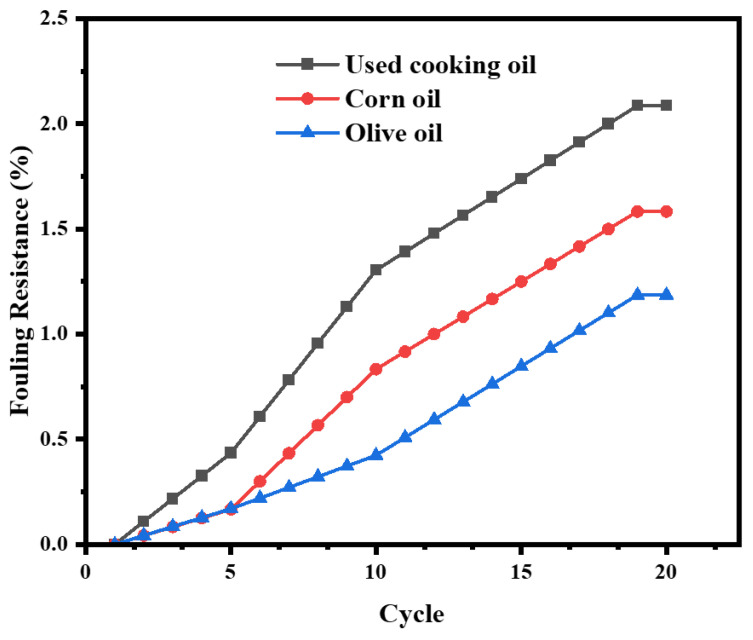
Fouling resistance vs. number of cycles for different oils.

This slight decline in flux is attributed to moderate oil accumulation within the membrane′s microchannels, particularly with used cooking oil which has a more complex chemical composition. However, the fouling resistance (*R_F_*)—defined as the flux loss due to irreversible membrane fouling—remained low, with maximum values of 2.09%, 1.58%, and 1.19% for used cooking oil, corn oil, and olive oil, respectively. Importantly, a simple rinse with an equimolar mixture of water and ethanol fully restored the membrane performance, effectively removing residual oil without leaving any deposits (Supplementary File Video S5). Throughout all 20 cycles, the final flux values remained high with minimal fouling resistance (*R_F_* < 3%), indicating the membrane′s stability and durability as shown in Figure 13.

The separation efficiency stayed high, exceeding 98% for all oils, with average values of 98.8% for corn oil and 98.6% for olive oil, demonstrating the membrane′s ability to maintain stable performance after repeated use.

## 4. Conclusions

In this study, a high-performance bilayer ecological membrane (BDLM) was developed from balsa wood, a biomass-derived material. Its heterostructured bilayer design, consisting of a carbonized transverse section infused with PDMS and a natural longitudinal section, optimized separation properties. The BDLM exhibited an average filtration rate of 1176.86 Lm^−2^ h^−1^ and a separation efficiency of 98.60% for various oils (corn oil, olive oil, and used cooking oil), with a mechanical strength of 10.92 MPa and an elongation at break of 10.42%. Additionally, BDLM demonstrated excellent fouling resistance and good reusability after multiple cycles. Its heterostructured design also enabled the separation of various organic solvents, oils, and even certain engine oils through a simple adsorption mechanism. Furthermore, thermal conductivity analysis indicated that this bilayer design could be used for heat-assisted separation, particularly solar evaporation for water treatment. These findings open promising prospects for the development of eco-friendly separation technologies based on renewable and biodegradable materials, without relying on toxic compounds or particles. Future research could focus on scaling up performance, exploring industrial applications, and extending its use to water decolorization and photothermal-assisted separation, providing sustainable and cost-effective environmental solutions.

## Figures and Tables

**Table 1:** An evaluation comparing the effectiveness of various membrane materials in repeated oil–water separation tests.

Types of Membranes	Permeate Flux (Lm^−2^ h^−1^)	Separation Efficiency (%)	Cycles	Reference
BDLM	1176	98.60	20	This work
ePTFE/fPTFE	208	98	12	[47]
M-PD/HPA	149.8	82.60	-	[48]
PVDF@FCC-MCF	650	99.00	12	[20]
PVP-VTES	17.45	91.47	-	[49]
Janus Wood	810	>99.6	-	[50]
Functionalized wood	>650	>99.2	30	[51]
Walnut wood	1300	98.5	3	[52]
Wood/MIL-100(Fe)	1061	99	10	[53]

## Data Availability

The data that support the findings of this study are available from the corresponding author upon reasonable request.

## Supplementary Materials

The following supporting information can be downloaded at: https://www.mdpi.com/article/10.3390/membranes15060160/s1.
